# Increasing Representation of Black Primary Care Physicians—A Critical Strategy to Advance Racial Health Equity

**DOI:** 10.1001/jamanetworkopen.2023.6678

**Published:** 2023-04-03

**Authors:** Monica E. Peek

**Affiliations:** The University of Chicago, Section of General Internal Medicine, Center for the Study of Race, Politics and Culture, Chicago, Illinois.

Racial disparities in health care and health outcomes have been perniciously difficult to eradicate despite local, regional, and national efforts to address them over the past several decades. Strategies have primarily focused on increasing health care access (eg, Medicaid expansion, the Affordable Care Act) and quality (eg, patient-centered care), with recent efforts promoting the integration of social care (ie, addressing patients’ social needs such as food insecurity) into comprehensive treatment plans.^1^

Increasing the number of physicians who are racial and ethnic minority individuals has been another strategy to improve clinical care delivery and health outcomes among minoritized racial and ethnic populations. The evidence associating physician race with patient health outcomes has primarily been limited to research interventions and/or clinical settings. For example, Cooper et al^2^ found that Black patients in racially concordant physician relationships had higher levels of positive physician affect, were more satisfied with their health care, and rated their physicians as more participatory in decision-making. Saha et al^3^ reported that Black patients who had Black physicians were more likely to report having preventive care services and “all needed medical care” during the prior year. However, the association between Black physicians and the health of minoritized racial groups and health disparities at the population level has not been previously evaluated.

In *JAMA Network Open*, Snyder et al^4^ used longitudinal data at the discrete time points of 2009, 2014, and 2019 from 1618 US counties that had at least 1 Black primary care physician (PCP) and measured the associations between the representation of Black PCPs and the mortality and survival rates both within counties and between counties. The authors’ definition of Black PCP representation is important because it is accurate regardless of changes to the size of the physician workforce or the population being served. The authors defined Black PCP representation as the ratio of the proportion of PCPs who identified as Black divided by the proportion of the population who identified as Black. The authors’ evaluation of between-county differences and within-county differences allowed the research team to explore variations that may be more likely due to changes over time (within-county variation) compared with inherent differences in place (between-county variation) that reflect infrastructure and resources needed to support healthy communities.

The authors found that a 10% increase in Black representation was associated with a 30.61-day increase in life expectancy for Black individuals (95% CI, 19.13–42.44 days), a reduction in all-cause mortality among Black persons by 12.71 deaths per 100 000 (95% CI, −14.77 to −10.66), and a 1.17% reduction in the Black/White disparity in all-cause mortality (95% CI, −1.29% to −1.05%). The associations with life expectancy were strongest in counties with high rates of poverty. During a given year of heightened Black representation within counties (vs their average), there were reduced mortality rates among Black populations (−35.34 [95% CI, −58.86 to −11.81] deaths per 100 000 ) and smaller Black/White disparities in all-cause mortality (−2.44 [95% CI, −3.65 to −1.23]).

This study’s findings are important for several reasons. First, they demonstrate that at a population level, mortality and life expectancy among Black individuals are improved when there is greater representation of Black PCPs within the community—a representation that aligns more closely with that of the population. These health outcomes are not exclusively related to health care delivery and use. While there is evidence to support potential mechanisms by which Black physicians working within the health care system can improve health outcomes for Black patients (eg, increased shared decision-making and patient-centered care, culturally concordant care, increased quality of care), there is also evidence that Black physicians are more likely than physicians from other racial or ethnic groups to engage in health-related work outside the health care system—that is, Black physicians are more likely to provide health-related expertise to local community organizations (eg, school boards, local media); to be politically involved in health-related matters at the local, state, or national level; and to encourage medical organizations to advocate public health (eg, air pollution, gun control, increased literacy, substance abuse prevention).^5^ This community involvement and advocacy by Black physicians may change the social drivers of health for the populations most vulnerable to their health effects.

Second, the study’s mortality associations were more pronounced in counties with higher rates of poverty. In addition to factors noted above, this finding may also reflect that Black physicians disproportionately care for patients that are uninsured and underinsured compared with their non-Hispanic White counterparts. In a study of factors associated with PCPs’ decisions to accept new Medicaid patients under Michigan’s Medicaid expansion, Tipirneni et al^6^ found that Black physicians had an adjusted odds ratio of 3.46 (95% CI, 1.45–8.25) of accepting new Medicaid patients, more than any other racial or ethnic group within the physician workforce.

Third, no significant associations were found between the total proportion of PCPs and life expectancy of Black populations or mortality rates among Black populations, although there was a small decrease in the Black-White mortality disparity rate. This not only underscores the importance of Black physicians to the health and well-being of Black patients, but points to the continued chasm between non-Black physicians and Black patients that has been created by generations of structural racism, medical experimentation and other abuses, clinician bias, and subsequent patient distrust and disengagement.^7^ Overcoming this chasm, establishing trustworthy institutions, and engaging with and truly seeing Black patients in their full humanity will take an extraordinary, transformative, and sustained commitment of time, infrastructure, and action.

Last, more than 50% of US counties were ineligible at each of the 3 study time points because they did not have a single Black PCP in the entire county. During the same study period, 90.9% to 94.1% of US counties had at least 1 physician of any race or ethnicity. In addition, none of the counties had proportions of Black PCPs that were equivalent to the proportion of Black individuals in the population. Given the extraordinary association between Black PCP representation and population outcomes for Black communities, it should be a national priority to ensure that Black populations in the US have access to Black PCPs within their counties and to increase the representation of Black PCPs within existing counties.

This study has brought to light the importance of Black PCP representation to public health outcomes among Black populations across the US. Increasing this representation must become a multifaceted national strategy to improve health and increase equity among Black populations in the US.
