# Errors in Commentary

**DOI:** 10.1001/jamanetworkopen.2023.17327

**Published:** 2023-05-22

The Invited Commentary “Increasing Representation of Black Primary Care Physicians—A Critical Strategy to Advance Racial Health Equity,”^1^ published April 14, 2023, mischaracterized some elements of the Original Investigation “Black Representation in the Primary Care Physician Workforce and Its Association With Population Life Expectancy and Mortality Rates in the US” by Snyder et al.^2^ The commentary indicated that the total number of counties studied was 1168; it should have been given as 1618. The commentary stated that “no significant associations were found between the total proportion of PCPs and population outcomes for Black communities.” It should have read “no significant associations were found between the total proportion of PCPs and life expectancy of Black populations or mortality rates among Black populations, although there was a small decrease in the Black-White mortality disparity rate.” In addition, the commentary stated that “more than 50% of US counties were ineligible for this study” when it is more accurate to say that “more than 50% of US counties were ineligible at each of the 3 study time points.” This article has been corrected.
